# A Novel Trimethylamine Oxide-Induced Model Implicates Gut Microbiota-Related Mechanisms in Frailty

**DOI:** 10.3389/fcimb.2022.803082

**Published:** 2022-03-11

**Authors:** Si-yue Chen, Xing-yu Rong, Xin-yi Sun, Yi-rong Zou, Chao Zhao, Hui-jing Wang

**Affiliations:** ^1^ Graduate School, Shanghai University of Traditional Chinese Medicine, Shanghai, China; ^2^ Laboratory of Neuropsychopharmacology, College of Fundamental Medicine, Shanghai University of Medicine & Health Science, Shanghai, China; ^3^ Key Laboratory of Medical Molecular Virology (MOE/NHC/CAMS), School of Basic Medical Sciences, Shanghai Medical College, & National Clinical Research Center for Aging and Medicine, Huashan Hospital, Fudan University, Shanghai, China; ^4^ Shanghai Frontiers Science Center of Pathogenic Microbes and Infection, Shanghai, China

**Keywords:** frailty, TMAO, gut barrier, gut microbiota, serum metabolomics

## Abstract

Frailty is a complicated syndrome that occurs at various ages, with highest incidence in aged populations, suggesting associations between the pathogenesis of frailty and age-related changes. Gut microbiota (GM) diversity and abundance change with age, accompanied by increased levels of trimethylamine oxide (TMAO), a systemic inflammation-inducing GM metabolite. Thus, we hypothesized that TMAO may be involved in the development of frailty. We successfully established and verified a novel model of frailty in adult mice based on a 4-week intraperitoneal injection regime of TMAO followed by LPS challenge. The frailty index significantly increased in TMAO-treated mice after LPS challenge. TMAO also decreased claudin-1 immunofluorescent staining intensity in the jejunum, ileum, and colon, indicating that the destruction of intestinal wall integrity may increase vulnerability to exogenous pathogens and invoke frailty. 16S sequencing showed that TMAO significantly reduced the GM Firmicutes/Bacteroidetes (F/B) ratio, but not α-diversity. Interestingly, after LPS challenge, more genera of bacterial taxa were differently altered in the control mice than in the TMAO-treated mice. We infer that a variety of GM participate in the maintenance of homeostasis, whereas TMAO could blunt the GM and impair the ability to recover from pathogens, which may explain the continuous increase in the frailty index in TMAO-treated mice after LPS challenge. TMAO also significantly increased serum imidazole metabolites, and led to different patterns of change in serum peptide and phenylpropanoid metabolites after LPS stimulation. These changes indicate that glucose metabolism may be one mechanism by which GM inactivation causes frailty. In conclusion, TMAO leads to frailty by destroying intestinal barrier integrity and blunting the GM response.

## Introduction

Frailty is defined as a decrease in the reserves and recovery function of the body. Decreased physical and cognitive reserves lead to increased vulnerability to stressors (Mueller et al., 2016), and make individuals more susceptible to illness. Reserve capacity is a measure of the threshold of disease of the body. Frailty can also be explained by the accumulation of damage to various systems of the body over time. Frailty affects multiple organs and systems, namely, the skeletal muscles and immune, endocrine, hematopoietic, and cardiovascular systems (Fulop et al., 2015). Patients with frailty are vulnerable to comorbidities, such as neurodegenerative diseases (dementia), cardiovascular diseases (chronic heart failure, atherosclerosis), and type II diabetes (Fülöp et al., 2016). Frailty may be associated with age, but not necessarily with aging. According to an investigation by Xing et al. (Xi and Guo, 2014), 43.2% of 683 older people (≥60 years old) from Beijing did not have frailty, while the incidence of pre-frailty was 45.7%, and 11.1% of the cohort were frail. These data suggest that healthy aging could be achieved by preventing and improving frailty status. However, our current understanding of frailty is extremely inadequate, and there is no consensus on the mechanisms that lead to frailty or even the diagnostic criteria. These knowledge deficiencies prevent the design of appropriate interventions to achieve the goal of healthy aging.

Due to the boom in studies on the gut microbiota in recent years, the roles of the gut flora in aging and frailty have become increasingly reported. The gut microbiota is an extremely large, important component of the body that changes with age and health status, and affects many processes within the host. Studies (Salazar et al., 2017) indicate that aged adults have higher abundances of *Clostridum* Cluster XIVa, *Faecalibacterium prauanitzii*, *Actinomycetes* (mainly *Bifidobacterium*), and *Proteobacteria* than young adults. Rampelli et al. (2013) also reported that the abundance of *Faecalibacterium*, *Eubacterium*, and *Bifidobacterium* increased in the aged population.

In addition to the gut flora changing with age, there is also a correlation between the status of the body and the composition of the gut microbiota. Compared to a non-frail group, frail individuals had a higher abundance of *Eubacterium dolichum* and *Eggerthella lenta*, and a lower abundance of *F. prausnitzii* (Jackson et al., 2016). Based on FI_34_ values, *Eggerthella*, *Ruminococcus*, and *Coprobacillus* were reported to have the greatest impact on frailty (Maffei et al., 2017). According to the Rockwood Frailty Index, *F. prausnitzii* exhibited an inverse correlation with age, while *E. dolichum* and *E. lenta* exhibited positive correlations (Ticinesi et al., 2017). Frailty is often reflected by low biodiversity of the gut microbiota, decreased abundance of butyrate producers, and increased numbers of pathobionts (Casati et al., 2019). *Escherichia*, *Ruminococcus* (Rampelli et al., 2013), and *Clostridium leptum*, among others, are increased and *F. prausnitzii* and other species are decreased in centenarians (Biagi et al., 2010). Biagi et al. (2010) also showed that the proportion of *Clostridium* cluster XIV a was significantly lower in centenarians than in elderly and younger adults. Furthermore, the diversity and abundance of the intestinal microbiota vary between elderly adults and centenarians (Biagi et al., 2010; Rampelli et al., 2013). To a certain extent, these findings support the hypothesis that specific gut microbiota may contribute to healthy aging.

The gut microbiota may promote the processes of aging and frailty *via* different mechanisms. Direct effects could be accomplished *via* the gut microbiota itself or by its metabolites. For example, an increased abundance of *Clostridium* may elevate the levels of trimethylamine (TMA). Dietary choline, L-carnitine, and betaine can be metabolized to TMA by Firmicutes and Proteobacteria (Yang et al., 2019). TMA can be further converted to trimethylamine oxide (TMAO) by flavin (Yang et al., 2019). Given the increase in *Clostriduim* reported in older individuals, older adults may possibly have higher TMAO levels (Meyer et al., 2016), which may contribute to the incidence of frailty.

A link has previously been suggested between TMAO and frailty. A clinical investigation found frail individuals had significantly higher contents of TMAO than non-frail individuals (He et al., 2020). This finding also supports our hypothesis that TMAO is associated with frailty. TMAO could induce significant oxidative stress, inflammatory conditions, and endothelial dysfunction, as it can stimulate the TXNIP-NLRP3 inflammasome, activate release of the inflammatory cytokines IL-1β and IL-18, and also inhibit the production of endothelial nitric oxide synthase (eNOS) and nitric oxide (NO) (Li et al., 2017).

The gut microbiota and its metabolite TMAO may contribute to the occurrence and development of frailty. Thus, improving the function of the gut microbiota may provide a potential target to prevent frailty. To test this hypothesis, the present study first evaluated a TMAO-induced model of frailty in mice. Then, we determined and analyzed the changes in the gut microbiota and serum metabolites, and used this model to investigate the mechanisms by which the gut flora is related to frailty, independently of aging. Though the process of aging cannot be avoided, amelioration of frailty provides a potential strategy to achieve healthy aging.

## Materials and Methods

### Animals

Male C57BL/6 mice aged 10–14-weeks-old were maintained in the animal facility of the Shanghai University of Medicine & Health Science (SUMHS). All experiments were performed in accordance with international guidelines, were approved by the animal ethics committee of SUMHS, and were performed in a blinded manner.

### TMAO-Induced Frailty Model and LPS Challenge

The animals were randomly assigned to four groups: control + saline, TMAO + saline, control + LPS, or TMAO + LPS (*n* = 8 per group).

To induce TMAO-induced frailty, mice were intraperitoneally injected with TMAO (trimethylamine N-oxide; Sigma-Aldrich, MI, USA; 0.16 mg/kg, dissolved in saline) every day for 4 weeks. The mice in the control group were injected with equivalent volumes of saline. After 4 weeks of TMAO/control treatment, LPS (lipopolysaccharide from *Escherichia coli* O111: B4, Sigma-Aldrich; 5 mg/kg, dissolved in saline) was administrated intraperitoneally once to challenge the mice. An equivalent volume of saline was injected as a vehicle control.

### Ethical Approval Statement

This animal study was reviewed and approved in advance by the animal ethics committee of the Shanghai University of Medicine & Health Science (No. 2019-GJZDYFJH-08-054629).

### Frailty Index

The frailty index was calculated based on four parameters: weight loss, weakness (grip strength), slow walking speed (Rota rod test), and exercise endurance (Rota rod test). The grip strength test was performed following the instructions of the IITC Life Science Grip Strength Meter Operating Manual (IITC Life Science, Woodland Hills, CA, America) (Gomez-Cabrera et al., 2017), and the Rota rod test for walking speed, in accordance with the IITC Life Science Rotarod Mice/Rats Operating Manual (IITC Life Science). Each test started at 4 rotations per minute (rpm), and then accelerated to 40 rpm over 300 s. The total test time was 350 s. The speed at which the mice fell was recorded. Three tests were performed with 10-min rest intervals between each test. The Rota rod test for exercise endurance was performed at a constant speed of 15 rpm over 600 s; the distance at which the mice fell was recorded.

The frailty index (FI) score was calculated as (FI_BW_ + FI_MS_ + FI_WS_ + FI_EE_)/4. **Table 1** summarizes the details used for each item included in the FI assessment.

**Table 1:** Details of the items in the improved frailty index score.

Item	Method	Score
Weight loss	Baseline body weight (BW) was measured on 3 consecutive days before TMAO treatment, and means and SD of each group were calculated. BW was measured every 2 days during the 4-week model; the change in BW was calculated for each mouse.	The FI_BW_ score for each mouse was defined as:
		0, if BW decreases ≦0
		0.25, if BW decreases <1/2 SD
		0.5, if BW decreases <1 SD
		0.75, if BW decreases <3/2 SD
		1, if BW decreases ≧3/2 SD
Weakness (muscle strength)	Baseline muscle strength (MS) was measured by the grip test on 3 consecutive days before TMAO treatment, and means and SD of each group were calculated. MS was measured every 2 days during the 4-week model; the change in MS was calculated for each mouse.	The FI_MS_ score for each mouse was defined as:
		0, if MS decreases ≦0
		0.25, if MS decreases <1/2 SD
		0.5, if MS decreases <1 SD
		0.75, if MS decreases <3/2 SD
		1, if MS decreases ≧3/2 SD.
Walking speed	Baseline walking speed (WS) was measured by the Rota rod test on 3 consecutive days before TMAO treatment, and the means and SD of each group were calculated. WS was measured every 2 days during the 4-week model; the change in WS was calculated for each mouse.	The FI_WS_ score for each mouse was defined as:
		0, if WS decreases ≦0
		0.25, if WS decreases <1/2 SD
		0.5, if WS decreases <1 SD
		0.75, if WS decreases <3/2 SD
		1, if WS decreases ≧3/2 SD.
Exercise Endurance	Baseline exercise endurance (EE) was measured by the Rota rod test on 3 consecutive days before TMAO treatment, and means and SD of each group were calculated. EE was measured every 2 days during the 4-week model; the change in EE was calculated for each mouse.	The FI_EE_ score for each mouse was defined as:
		0, if EE decreases ≦0
		0.25, if EE decreases <1/2 SD
		0.5, if EE decreases <1 SD.
		0.75, if EE decreases <3/2 SD
		1, if EE decreases ≧3/2 SD.

### Immunofluorescent Staining for Claudin-1

At the end of the experiment (72 h after LPS challenge or saline), mice were deeply anesthetized with an overdose of sodium pentobarbital and transcardially perfused with PBS, followed by 4% paraformaldehyde, after which the jejunum, colon, and ileum were collected. Tissues were post-fixed in 4% paraformaldehyde for 4 h, cryoprotected in 20% sucrose in PBS at 4°C, followed by 30% sucrose in PBS overnight, then embedded in OCT compound and frozen at −80°C. Frozen sections (14 mm) were washed with 0.01 M PBS followed by 0.03% PBS Triton X-100, incubated with 5% bovine serum albumin (BSA; VWR Life Science) for 2 h at room temperature, incubated overnight at 4°C with claudin-1 (D5H1D) XP^®^ Rabbit mAb (1:100, CST, Boston, Massachusetts, USA), washed with 0.03% PBST, and both subsequently incubated with Goat polyclonal Secondary Antibody to Rabbit IgG-H&L (Alexa Fluor^®^ 488) (1:1,000, Abcam Cambridge, UK). All staining procedures were performed in parallel for the different groups.

The sections were imaged with a DFC450 C fluorescent microscope (Leica Microsystems Ltd., Weltzar, Germany) using identical exposure times for all slides. Claudin 1 was quantified in at least three distantly separate sections of intestinal segments. The average background fluorescence for control staining sections was subtracted before calculation of the claudin-1 staining intensity using NIH ImageJ (Nahid et al., 2019).

### 16s rRNA Sequencing of the Gut Microbiota

Mouse fecal samples were collected at two timepoints: TMAO treatment after 4 weeks and 72 h after LPS injection.

Total nucleic acid was extracted from feces using the FastDNA^®^ SPIN Kit for Soil (MP Biomedicals, Irvine, CA, USA) according to the instructions of the manufacturer. The DNA purity and concentration of each sample were assessed using a NanoDrop2000 (Thermo Fisher Scientific, Wilmington, DE, USA). The quality of extracted DNA was determined by 1% agarose gel electrophoresis. The V3/V4 regions of the 16S rRNA gene were amplified by the PCR system with primers 338F (5`-ACTCCTACGGGAGGCAGCAG-3`) and 806R (5`-GGACTACHVGGGTWTCTAAT-3`) to further verify the quality of the DNA samples. The PCR system was performed in triplicate with 20 ml mixtures containing 4 ml of 5× FastPfu buffer, 2 ml of 2.5 mM deoxynucleoside triphosphates, 0.8 ml of each primer (5 mM), 0.4 ml of FastPfu polymerase, 10 ng of template DNA, and double-distilled water. PCR reactions were performed on an ABI GeneAmp^®^ 9700 (Thermo Fisher, Waltham, MA, USA) using the following cycling parameters: 95°C for 3 min, followed by 27 cycles at 95°C for 30 s, 55°C for 30 s, and 72°C for 45 s, with a final extension at 72°C for 10 min. The amplicons were extracted from a 2% agarose gel, purified using the AxyPrep DNA Gel Extraction Kit (Axygen Biosciences, Union City, CA, USA), and quantified by QuantiFluor™-ST (Promega, Madison, WI, USA) according to the instructions of the manufacturer. Equimolar quantities of purified amplicons were pooled and paired-end sequenced (2 × 300) on an Illumina MiSeq PE300 (Illumina) according to the specifications of the manufacturer for the MiSeq Reagent Kit v3.

The raw Illumina read data for 16S sequencing were deposited into the database of the National Health Institute with accession number PRJNA778423.

### Targeted Serum Metabolomics

Serum samples were collected at the end of the experiment (72 h after LPS challenge or saline) and stored at −80 ℃. The metabolomics analysis was performed on a Q300 Kit (Metabo-Profile, Shanghai, China) according to the instructions of the manufacturer. Briefly, 25 µl serum samples were added to 96-well plates, derivatized at 30°C for 60 min, evaporated for 2 h, 330 µl of ice-cold 50% methanol solution was added to reconstitute the sample, the plates were incubated at −20°C for 20 min, centrifuged at 4,000×*g* for 30 min at 4°C, and 135 µl of supernatant was transferred to a new 96-well plate with 10 µl of internal standard in each well. Serial dilutions of derivatized stock standards were added to the plate in separate wells and the plate was sealed for LC–MS analysis. All standards were obtained from Sigma-Aldrich (St. Louis, MO, USA), Steraloids Inc. (Newport, RI, USA), or TRC Chemicals (Toronto, ON, Canada), and accurately weighed and prepared in water, methanol, sodium hydroxide, or hydrochloric acid to obtain individual 5.0 mg/ml stock solutions. Appropriate amounts of each stock solution were mixed to create stock calibration solutions.

Ultraperformance liquid chromatography coupled to tandem mass spectrometry (UPLC-MS/MS) was performed on an ACQUITY UPLC-Xevo TQ-S system (Waters Corp., Milford, MA, USA) by Metabo-Profile Biotechnology (Shanghai) Co., Ltd. The optimized instrument settings for HPLC were an ACQUITY HPLC BEH C18 1.7 × 10^−6^ m VanGuard precolumn (2.1 × 5 mm) and ACQUITY HPLC BEH C18 1.7 × 10^−6^ m analytical column (2.1 × 100 mm), column temperature: 40°C, sample manager temperature: 10°C, mobile phases: A = water with 0.1% formic acid and B = acetonitrile/IPA (70:30), gradient conditions: 0–1 min (5% B), 1–11 min (5–78% B), 11–13.5 min (78–95% B), 13.5–14 min (95–100% B), 14–16 min (100% B), 16–16.1 min (100–5% B), and 16.1–18 min (5% B), flow rate: 0.40 ml min^−1^, and injection vol.: 5.0 µl. For mass spectrometry, capillary: 1.5 (ESI+), 2.0 (ESI−) Kv, source temperature: 150°C, desolvation temperature: 550°C, and desolvation gas flow: 1,000 L/h.

The raw data files generated by UPLC-MS/MS were processed using the iMAP platform (v1.0; Metabo-Profile, Shanghai, China). Principal component analysis (PCA) and orthogonal partial least squares discriminant analysis (OPLS-DA) were also performed using the iMAP platform. VIP (variable importance in projection) was obtained based on the OPLS-DA model. Metabolites with a VIP of ≥1 and *p*-value <0.05 (univariate analyses were based on whether the data were normally distributed) were regarded as statistically significant. The Z-score indicates how many standard deviations an observation is above or below the mean of the control group. Vplots were plotted to integrate the fold change and *p*-values were used to depict the significantly different metabolites.

### Statistical Analysis

Data are expressed as mean ± SEM. For FI assessment and claudin-1, statistical analysis was carried out using one-way or two-way ANOVA followed by Bonferroni analysis with GraphPad Prism software. For 16S bacterial abundance and targeted serum metabolomics analysis, the Wilcoxon rank-sum test in R (http://www.R-project.org/), was used for statistical comparisons. A *P*-value of less than 0.05 was considered statistically significant.

## Results

### Establishment of a TMAO-Induced Mouse Model of Frailty

**Figure 1:**
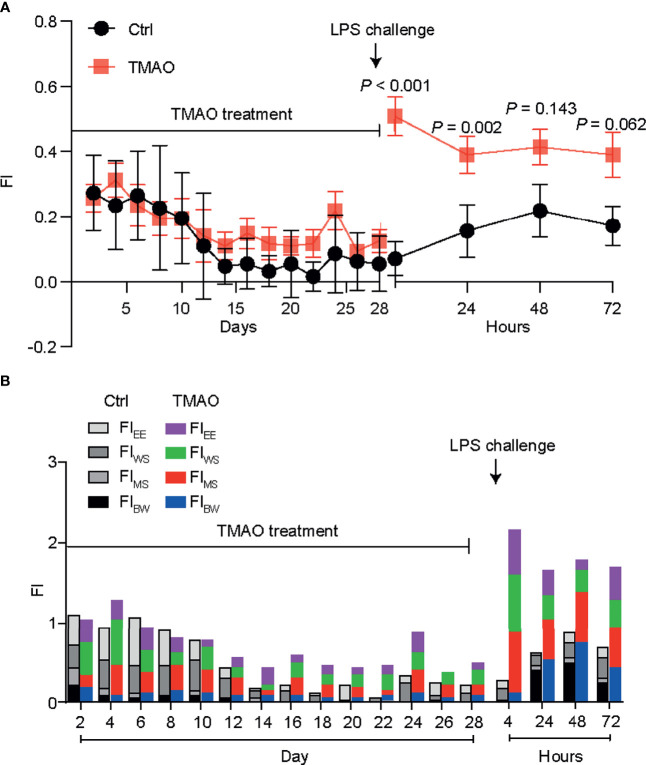
**(A)** Changes in the frailty index in the TMAO-induced frailty model. Mice were treated with TMAO for 28 days then subjected to LPS challenge for 72h. The frailty index is based on four behavioral tests. Control group (black line), TMAO group (red line). **(B)** Changes in four indicators that measure changes in frailty index. Values are presented as mean ± SEM of 8 mice per group.

**Figure 1A** shows the changes in the frailty index (FI) in the mouse model of TMAO-induced frailty. The total FI was calculated using four behavioral parameters, as described in **Table 1**; a higher FI indicates more severe frailty. After intraperitoneal injection of TMAO for 4 weeks, the FI of the TMAO-treated group was slightly higher than that of the control group injected with saline, though this difference was not significant (*p >*0.05). However, compared to the control group, the FI of the TMAO group increased rapidly and significantly at 24 h after the administration of LPS. Moreover, the FI of the TMAO group remained higher than the FI of the control group at 72 h after LPS challenge. Furthermore, the proportions of the four-item FI values are shown in **Figure 1B**. The data show that these four items contributed to the total FI values, indicating that the assessment of frailty should be evaluated by multiple indexes, not by a single one. The indexes selected in the present study not only simplify the operation process, but are also discriminative.

These changes match well with the characteristics of frailty in humans: although the FI did not obviously change after the 4-week administration of TMAO, the ability of the TMAO-treated mice to cope with an acute stressor (like LPS challenge) was significantly impaired. Thus, the intraperitoneal injection of TMAO for 4 weeks successfully established a mouse model of frailty.

### TMAO Disrupts Intestinal Barrier Integrity

**Figure 2:**
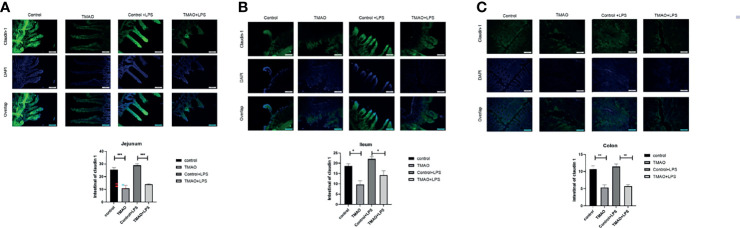
Changes in claudin-1 expression for intestinal barrier integrity. Changes in claudin-1 expression in the **(A)** jejunum, **(B)** ileum, and **(C)** colon in the TMAO-induced frailty model. Claudin-1 expression was quantified by immunohistochemical staining at 72 h after LPS challenge. The value for the control group was set to 100% and the changes in claudin-1 expression in the other groups are normalized to the control group. *p <0.05, **p <0.01, ***p <0.005, One-way ANOVA.

The integrity of the intestinal barrier is critical for protecting the body against pathogens or harmful substance. Thus, intestinal barrier integrity was assessed by immunohistochemical staining for claudin-1, which is a common marker protein of intestinal wall integrity (**Figure 2**). After a 4-week intraperitoneal injection regime of TMAO, the florescence intensities of claudin-1 were significantly decreased in the jejunum, ileum, and colon compared to the control group. Quantitative analysis revealed that the administration of TMAO decreased claudin-1 expression by about 50% in the ileum and colon, and by nearly 60% in the jejunum (**Figures 2A–C**). However, in both the control mice and TMAO-treated mice, claudin-1 florescence intensities did not obviously change after administration of LPS (**Figures 2A–C**).

Overall, these data suggest that TMAO destroyed the integrity of the intestinal barrier, but that one dose of LPS did not further affect the integrity of the intestinal barrier.

### Changes in the Gut Microbiota After LPS Challenge in the TMAO-Induced Mouse Model of Frailty

**Figure 3:**
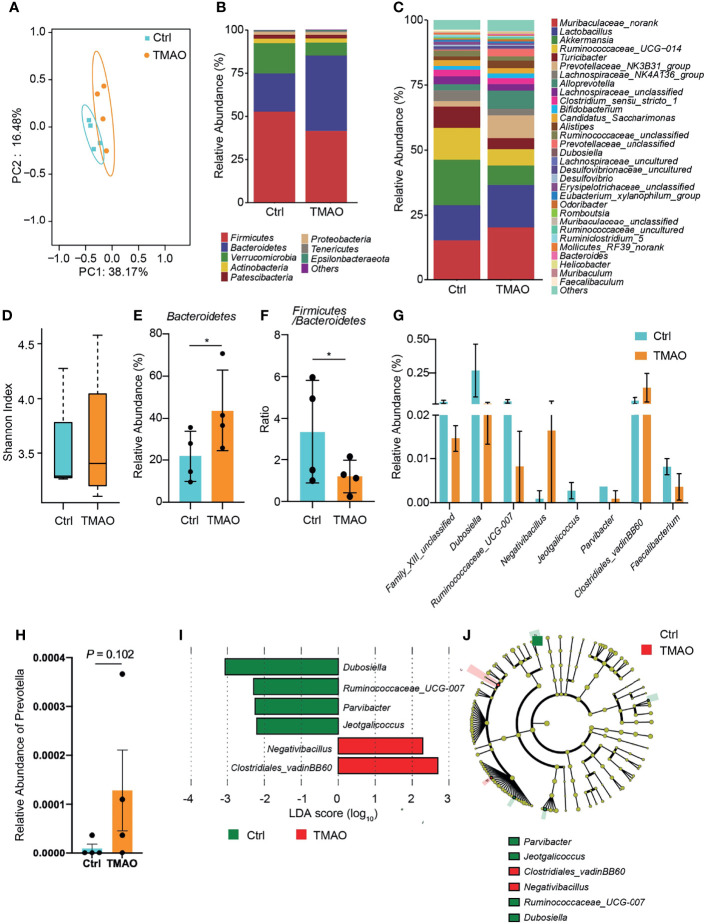
Changes in the gut microbiota in the TMAO-induced frailty model. Fecal samples were collected after 4-week administration of TMAO. **(A)** Graphic PCoA for the control group (blue squares) and TMAO group (orange squares) at the operational taxonomic unit (OTU) level. **(B)** Average relative abundance of prevalent microbiota at the family level in the control and TMAO groups. **(C)** Average relative abundance of prevalent microbiota at the genus level in the control and TMAO groups. **(D)** Shannon indices of alpha-diversity of the gut microbiota in the control group (blue squares) and TMAO group (orange squares). **(E)** Relative abundance of Bacteroidetes, expressed as (%), was significantly lower in the control group (blue) than TMAO group (orange). **(F)** The Firmicutes/Bacteroidetes ratio was lower in the TMAO group (orange) than control group (blue). **(G)** Boxplot of the top eight differentially enriched genera in the control group (blue) and TMAO (orange) group. **(H)** Relative abundance of *Prevotella* was increased in the TMAO group (orange) than in the control group (blue) **(I)** LEfSe analysis showing bacterial taxa with significantly different abundances between the control and TMAO group. Green (red) indicates increased (decreased) abundance in the control group. **(J)** Taxonomic cladogram obtained from LEfSe analysis showing the distribution of bacterial taxa (phylum, class, and order) that were differentially abundant in the control and TMAO groups. Green (red) squares indicate increased abundance (decreased) in the control group. *p <0.05, Wilcoxon rank-sum test.

To investigate the gut microbiota changes associated with TMAO-induced frailty, the intestinal microbial species were classified and analyzed by 16S ribosomal RNA gene sequencing. The Shannon index and principal co-ordinate analysis (PCoA) were similar between the TMAO group and control group (**Figures 3A, D**), suggesting bacterial diversity was maintained after TMAO treatment.

However, the composition of the gut microbiota was altered by TMAO (**Figures 3B, C**). Compared to the control group, at the phylum level, the proportion of Bacteroidetes lineages remarkably increased (by 100%) after TMAO administration (**Figures 3B, E**). The proportion of Firmicutes decreased slightly, although there was no significant difference between groups, and the Firmicutes/Bacteroidetes (F/B) ratio was significantly lower in the TMAO group than in the control group (*p <*0.05, **Figure 3F**). A decreased F/B ratio has previously been correlated with frailty (Kim and Jazwinski, 2018; Grigor’eva, 2020; Afonso et al., 2021). The major significantly altered genera are listed in **Figures 3G, I, J**.

Considering the significant changes of imidazole metabolites in the following metabolomics study, we checked the changes of Prevotella, an imidazole producer, after TMAO treatment. We found that after TMAO treatment, *Prevotella* increased with a *p*-value ≈ 0.1 (**Figure 3H**). This increasing trend may suggest the possible mechanism of TMAO treatment changing Prevotella to increase imidazole products and eventually lead to frailty.

**Figure 4:**
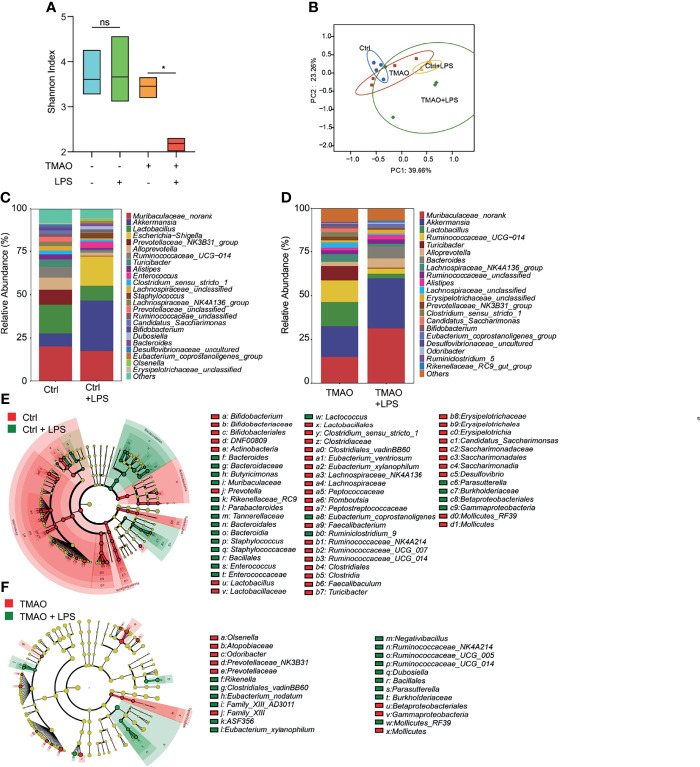
Changes in the gut microbiota after LPS challenge in the TMAO-induced frailty model. Serum samples were collected after 4-week administration of TMAO or 72 h after LPS challenge. **(A)** Shannon indices of gut microbiota alpha-diversity in the control (blue), control + LPS (green), TMAO (orange), and TMAO + LPS (red) groups. **(B)** Graphic PCoA of the control (blue dots), control + LPS (yellow triangles), TMAO (red squares), and TMAO + LPS groups (green diamonds) at the OTU level. **(C)** Average relative abundance of prevalent microbiota at the genus level in the control and control + LPS groups. **(D)** Average relative abundance of prevalent microbiota at the genus level in the TMAO and TMAO + LPS groups. **(E)** Taxonomic cladogram obtained from LEfSe analysis showing the distribution of bacterial taxa (phylum, class, and order) that were differentially abundant in the control and control + LPS groups. Green (red) indicates increased abundance in the control group (control + LPS group). **(F)** Taxonomic cladogram obtained from LEfSe analysis showing the distribution of bacterial taxa (phylum, class, and order) that were differentially abundant in the TMAO and TMAO + LPS groups. Green (red) indicates increased abundance in the TMAO group (TMAO + LPS group). *p <0.05, Ns indicates not significant, Wilcoxon rank-sum test.

Moreover, striking results were observed after LPS challenge in the TMAO-induced model of frailty. The Shannon index indicated that the gut flora of the control group still maintained high diversity after LPS administration, while the diversity decreased in the TMAO group (**Figure 4A**). PCoA analysis (**Figure 4B**) showed that LPS converted the microbiota in both the control and TMAO groups, with a large alteration in the gut microbiota of the control group, but a comparatively smaller change in the gut microbiota of the TMAO group. At the genus level, 22 genera decreased and 41 genera increased in the control mice after LPS challenge (**Figure 4E**). While, only 10 genera decreased and 15 genera increased in the TMAO-treated mice after LPS challenge (**Figure 4F**).

These data suggest that diverse bacteria may be needed in the active self-regulation mechanism within the gut microbiota in normal mice to maintain the stability of the internal environment. TMAO treatment reduced the capability of the response of the microbiota to the external challenges.

### Changes in Serum Metabolites After LPS Challenge in the TMAO-Induced Mouse Model of Frailty

**Figure 5:**
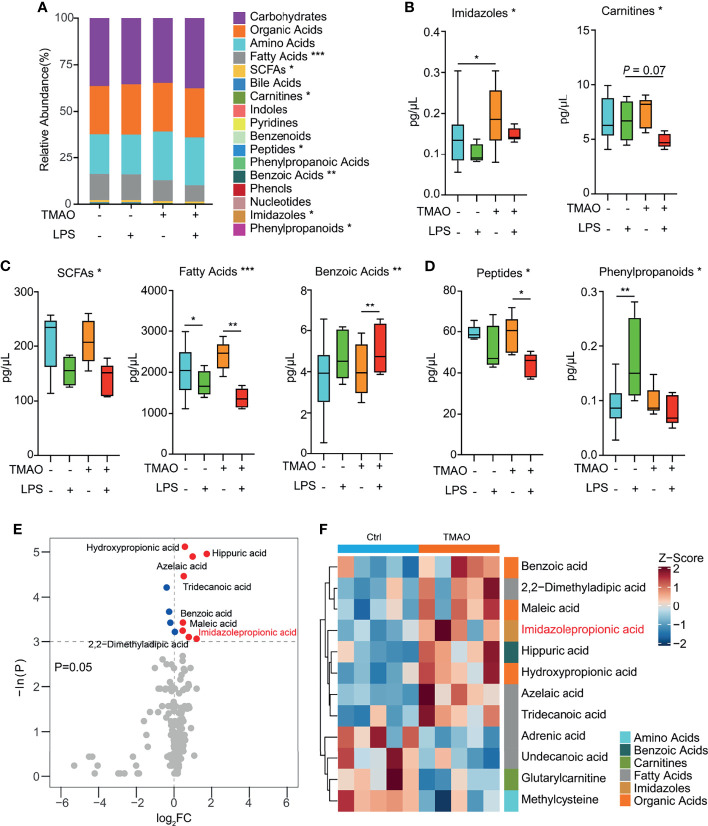
Changes in serum metabolites after LPS challenge in the TMAO-induced frailty model. **(A)** Average quantitative abundance of serum metabolites in the control, control + LPS, TMAO, and TMAO + LPS groups. **(B)** TMAO significantly induced imidazoles and carnitines. **(C)** The stress response to LPS significantly induced SCFAs, fatty acids, benzoic acids, and **(D)** differently altered peptides and phenylpropanoids. **(E)** Volcano map showing the differential metabolites identified by unidimensional statistical analysis. Metabolites colored blue (red) were reduced (increased) in the TMAO group compared to the control group. **(F)** Heatmap of the top 12 differently altered serum metabolites in individual mice, showing that the differences in the metabolite levels were associated with the experimental groups. *p <0.05, **p <0.01, ***p <0.005, Wilcoxon rank-sum test.

A targeted metabolomics study (**Figure 5A**) revealed that numerous metabolites, namely, fatty acids, SCFAs, peptides, benzoic acids, imidazoles, and phenylpropanoids were significantly altered in the serum of the TMAO-treated mice. Pairwise comparisons revealed that TMAO increased the levels of imidazoles (**Figure 5B**), and the administration of LPS decreased the levels of SCFAs, fatty acids, and benzoic acids in both the control and TMAO-treated groups (**Figure 5C**). However, peptides were not significantly different between the control and TMAO groups, but decreased significantly after LPS challenge in TMAO-treated mice. Phenylpropanoids increased in the control mice after LPS challenge, but did not change significantly in the TMAO-treated mice after LPS stimulation (**Figure 5D**).

Thus, the serum metabolomic analysis suggests imidazoles are a major class of metabolites relevant to TMAO-induced frailty. Analysis of specific imidazole metabolites (**Figures 5E, F**) revealed that imidazolepropionic acid was the only imidazole that changed significantly after TMAO treatment.

## Discussion

Due to the complexity of frailty, numerous criteria are used to evaluate frailty in scientific studies. The Fried score is a detailed listing of 24 indicators often used as a reference (Gomez-Cabrera et al., 2017), and includes seven items for the study, namely, integument, physical/musculoskeletal, vestibulocochlear/auditory ocular, neurological, digestive/urogenital, and respiratory. In the present study, four parameters based on different dimensions of frailty were chosen to calculate the FI. The original Fried score rules were refined to make them more discriminative. Moreover, the tests can be completed in an appropriate period of time, which reduces individual differences between experimental animals due to prolonged durations of testing, and thus makes the data more reliable.

We established a novel TMAO-induced frailty model in mice and applied this modified FI evaluation. A slight rise in the FI was observed after 4-week administration of TMAO, though this change was not statistically significant. Interestingly, subsequent challenge with LPS induced a strong, long-lasting increase in the FI in TMAO-treated mice. This phenotype closely resembles the definition of frailty, as individuals with frailty are rapidly weakened when challenged by causative factors and have greater difficulty recovering; this may be due to a decline in the recovery and self-regulation abilities of the frail body.

TMAO has been demonstrated to be involved in the aging process (Ke et al., 2018), as it affects vascular aging and endothelial cell senescence by decreasing cell proliferation, increases the expression of senescence markers to promote stalling in the G0/G1 phase of the cell cycle, and impairs cell migration. Singh et al. (2019) showed that TMAO significantly reduced the expression of the tight junction proteins ZO-2, VE-cadherin, and occludin in endothelial cell monolayers. Consistent with these reports, in the present study, TMAO was confirmed to cause defects in the integrity of the intestinal epithelium. TMAO significantly decreased the expression of claudin-1. Imidazolepropionic acid was previously reported to suppress the expression of tight junction-related genes, including claudin (Yoshimoto et al., 2021). Interestingly, in the present study, we observed an increase in the serum metabolite imidazolepropionic acid in the TMAO-treated mice and a decreased expression of claudin-1. Based on these reports, our data indicate that increased levels of imidazolepropionic acid after TMAO treatment contributed to the destruction of intestinal barrier integrity in our novel mouse model of frailty. This may be one of the reasons why TMAO leads to frailty in the model mice; however, the detailed molecular mechanisms need to be investigated further. Studies have indicated that TMAO may impair NO production by downregulating sirtuin 1 (SIRT1) expression and increase oxidative stress by causing endothelial dysfunction and activating endothelial cell senescence. An impaired intestinal barrier can induce systemic inflammation, and thus may play a powerful role in the development of metabolic diseases and result in decreased muscle strength and physical function, leading to frailty (Bhattacharya et al., 2020). Kühn et al. confirmed a correlation exists between intestinal wall integrity and frailty from another perspective. The gut brush border enzyme intestinal alkaline phosphatase (IAP) can inhibit inflammatory mediators and positively regulate intestinal barrier function and microbial homeostasis. Interestingly, IAP supplementation significantly reduced age-related gut permeability and gut-derived systemic inflammation, resulting in lower levels of frailty and an extended lifespan (Kühn et al., 2020).

Increased levels of TMAO in the body lead to systemic inflammation, and may further change the composition and function of the gut microbiota. For example, higher TMAO producers had more significantly elevated F/B ratios (Cho et al., 2017), similar to the changes observed in the intestinal flora of healthy elderly individuals (Vaiserman et al., 2020). The F/B ratio can indicate the state of the human body. For example, the F/B ratio is commonly used as a biomarker of obesity, and there is a positive association between frailty and obesity (Grigor’eva, 2020; Afonso et al., 2021). Moreover, the F/B ratio has recently been shown to be a biomarker of gut dysbiosis (Grigor’eva, 2020); thus, the F/B is a sign of gut health. Gut dysbiosis can trigger the innate immune response and chronic low-grade inflammation (Ke et al., 2018), which can promote the progression of frailty. In our model, the F/B ratio reduced after 4-week TMAO administration, which suggests that the TMAO-treated mice were already in a frail state, even though the FI had not significantly changed. After subsequent LPS challenge, the TMAO-treated mice exhibited severe, long-lasting frailty.

Interestingly, previous studies have shown that the diversity of the gut flora is clearly associated with aging and/or frailty. The complex intercommunication between the composition of the gut microbiota confers protection against pathogens, while also maintaining tolerance and balance of our body (Xu et al., 2018). Our data potentially offer more evidence for this theory. Normally, when the body encounters a causative stimulus—such as LPS in the present study—the gut microbiota composition changes significantly, indicating that high numbers of the gut microbiota are involved in the regulation of the body. However, in the TMAO-induced frailty state, significantly fewer gut microbiota was involved in the regulation of LPS-induced inflammation, which may limit stable, rapid recovery to organismal homeostasis. These data suggest that only maintaining the diversity of the gut microbiota composition is not sufficient to prevent or improve frailty, as the activities of the microbiota appear to be more important. Moreover, when the responses of the microbiota to changes in the micro-environment become blunted, it may become difficult to maintain and recover the balance of homeostasis, which may result in the phenotype of frailty. Therefore, we raise the suggestion that traditional diversity indexes may be not enough to judge the correlation between intestinal microbiota and frailty. Effective diversity, a new concept implied in the present study, which indicates the activities of intestinal microbiota, may be a potentially more closely related predictor.

The effects of the changes in the microbiota and disruption of intestinal wall integrity on frailty after TMAO treatment were reflected by changes in serum metabolites. The main class that changed significantly after TMAO treatment was imidazoles. Imidazoles can only be produced by certain gut microbiota, such as *Prevotella*_1, *Prevotellaceae*_UCG_003 (Liu et al., 2021), *Streptococcus mutans*, and *E. lenta* (Koh et al., 2018). As mentioned in our results, *Prevotella* was found to increase after TMAO treatment. This increasing trend suggests that the possible mechanism of TMAO treatment changed the abundance of *Prevotella*, increasing imidazole products and eventually leading to frailty. As discussed above, the increased levels of imidazolepropionic acid observed after TMAO treatment may contribute to the destruction of intestinal integrity indicated by claudin-1 staining in our novel mouse model of frailty (Yoshimoto et al., 2021). Furthermore, the increase in the levels of imidazoles indicates TMAO may affect glucose metabolism and further affect the energy supply of the body (Koh et al., 2018). The levels of SCFAs, fatty acids, and benzoic acids decreased in both the control groups and TMAO-treated groups after LPS challenge, indicating that these metabolites may be related to the acute stress response to LPS stimulation, rather than the frailty state induced by TMAO. Detailed analysis showed that imidazolepropionic acid primarily accounted for the change in imidazole metabolites. Imidazolepropionic acid has been reported as a metabolite of histidine (Koh et al., 2018). The literature shows that the imidazole moiety contributed by histidine residues is a ubiquitous functionality in proteins and peptides that is known to be involved in many binding and catalysis events (Buyst et al., 2015). This may also possibly explain why LPS-induced peptide metabolism changed more significantly in the TMAO-treated mice than in the control mice. However, further study is necessary to investigate how the changes in the levels of imidazoles affect other metabolites and, ultimately, lead to frailty.

In summary, we piloted and validated a novel mouse model of TMAO-induced frailty, and explored the gut microbiota mechanisms related to frailty. Imbalanced changes in the gut microbiota and the consequential alterations in the host imply a potential role for the gut microbiota to contribute to the process of frailty. As frailty is not necessarily a direct outcome of aging, this pilot study also indicates that regulation of the gut microbiota may provide a potential strategy to achieve healthy aging.

## Data Availability Statement

The original contributions presented in the study are publicly available in NCBI under accession number PRJNA778423.

## Ethics Statement

The animal study was reviewed and approved by the animal ethics committee of Shanghai University of Medicine and Health Science.

## Author Contributions

S-yC and X-yR designed the study, conducted the experiments, and drafted the manuscript. X-yS and Y-rZ conducted the animal behavior experiments. CZ and H-jW supervised the study, and contributed to writing and editing the manuscript. All authors listed have made a substantial, direct, and intellectual contribution to the work and approved it for publication.

## Funding

This work was supported by the National Key Research and Development Program of China (2018YFC2000500/03, 2018YFC2002000). CZ was supported by the original exploration program of Fudan University (2021).

## Conflict of Interest

The authors declare that the research was conducted in the absence of any commercial or financial relationships that could be construed as a potential conflict of interest.

## Publisher’s Note

All claims expressed in this article are solely those of the authors and do not necessarily represent those of their affiliated organizations, or those of the publisher, the editors and the reviewers. Any product that may be evaluated in this article, or claim that may be made by its manufacturer, is not guaranteed or endorsed by the publisher.
